# *Hdh-Tektin-4* Regulates Motility of Fresh and Cryopreserved Sperm in Pacific Abalone, *Haliotis discus hannai*

**DOI:** 10.3389/fcell.2022.870743

**Published:** 2022-04-25

**Authors:** Zahid Parvez Sukhan, Shaharior Hossen, Yusin Cho, Won Kyo Lee, Kang Hee Kho

**Affiliations:** Department of Fisheries Science, Chonnam National University, Yeosu, South Korea

**Keywords:** Tektin-4, sperm, motility, cryopreservation, Pacific abalone, *Haliotis discus hannai*

## Abstract

As structural components of sperm, tektins are thought to play a fundamental role in sperm flagellar motility. In this study, Tektin-4 (*Hdh-TEKT4*) gene was successfully cloned and characterized from the testis tissue in Pacific abalone, *Haliotis discus hannai*. The full-length cDNA of *Hdh-TEKT4* was 1,983 bp, with a coding region of 1,350 bp encoding 51.83 kDa putative protein of 449 deduced amino acids. *Hdh-TEKT4* contains a tektin domain including a nonapeptide signature motif (RPGVDLCRD). Fluorescence *in situ* hybridization revealed that *Hdh-TEKT4* localized in the spermatids of Pacific abalone testis. qRT-PCR analysis showed that *Hdh-TEKT4* was predominantly expressed in testis tissues. *Hdh-TEKT4* mRNA expression was upregulated during the fully mature testicular developmental stage in both seasonal development and EAT exposed abalone. Furthermore, mRNA expression of *Hdh-TEKT4* was significantly higher in sperm with higher motility than in sperm with lower motility during peak breeding season, induced spawning activity stages, and after cryopreservation in different cryoprotectants. Taken together, these results indicate that the expression of *Hdh-TEKT4* in Pacific abalone sperm might have a positive correlation with sperm motility.

## 1 Introduction

Sperm is the most divergent type of cell possibly because of its exposure to several physical, chemical, and environmental barriers in the outside of the body during active involvement in reproduction (Inaba, 2011). Spermatogenesis is a highly complex process of sperm cell development leading to the formation of functional spermatozoa for successful reproduction. It requires a coordination of both mitosis and meiosis cell divisions followed by cytoskeleton reorganization for reconstructing flagellated spermatozoa (Abe, 1987; Matsuyama et al., 2005). The fertility of sperm and successful reproduction largely depend on sperm motility (David et al., 2015; Hu et al., 2017). Sperm motility is generated by a highly organized, microtubule-based structure called the axoneme, which is composed of more than 250 proteins (Inaba, 2003). Tektins, an interesting family of axonemal proteins, have been reported in various mammals and invertebrates.

Tektins are structural components of sperm. They are thought to play a fundamental role in sperm ciliary, flagellar motility (Tanaka et al., 2004; Roy et al., 2007; Zuccarello et al., 2008), and spermatogenesis in humans (Xu et al., 2001) and abalone (Amparyup et al., 2010). So far, five tektin genes (Tektin-1 to Tektin-5) have been reported in mammals. Three Tektin genes, Tektin-A1, Tektin-B1, and Tektin-C1, orthologous to Tektin-4, Tektin-2, and Tektin-3 in mammals, respectively, have also been reported in invertebrate species (Amos, 2008). Tektin-1 is localized in the centrosome of spermatids and the caudal end of elongated spermatids. It might be involved in the nucleation of the flagellar axoneme. It is essential for assembly of the basal body (Larsson et al., 2000). Tektin-2 is localized in the axoneme of the sperm. The absence of Tektin-2 can lead to infertility of males, enhance flagellar bending, and reduce motility (Tanaka et al., 2004). Tektin-3 is present in the peri-axonemal component of flagella (Takiguchi et al., 2011). A deficiency of Tektin-3 in male does not hamper fertility or hyperactivation. However, it can reduce motility and forward progression and increase flagellar structural bending (Roy et al., 2009). Tektin-4 is located in the outer dense fibers and interacts with outer dense fiber-associated proteins (Iida et al., 2006). Males without Tektin-4 are subfertile with extreme reduction of ATP levels and forward progressive velocity (Roy et al., 2007). Tektin-4 is also involved in the transmission of the bending force from the axoneme through the outer dense fiber to the connecting piece. Tektin-5 gene is localized in the inner surface of the mitochondrial sheath in the sperm flagellar midpiece (Murayama et al., 2008). In abalone species, Tektin-4 and Tektin A1 (identical to Tektin-4) have been reported in *Haliotis discus discus* and *H. asinina* (GenBank Accession No. ARR97156.1 and ACJ15284.1), respectively.

Abalones are marine molluscan species distributed worldwide in tropical and temperate waters. Several abalone species including Pacific abalone (*Haliotis discus hannai*) have been found in Korea. Pacific abalone is considered the most commercially important species because of its popularity as food, high price, and extraordinary food value (Gordon and Cook, 2013; Sukhan et al., 2021a). However, the natural production of abalone in Korea has reduced greatly (Cook, 2016). Therefore, its commercial aquaculture was started in marine cages, which has now expanded extensively. All abalone aquaculture farms rear hatchery-produced abalone seeds for their production (Park and Kim 2013). The seed production of Pacific abalone in hatchery requires the best quality broodstock to uphold proper reproductive function. A number of exogenous and endogenous factors can affect the quality of broodstock. Among endogenous factors, the sperm quality of male abalone, especially sperm motility, is considered the most important. Sperm motility is necessary for the transport of male DNA to eggs in species with both external and internal fertilization (Inaba 2003). Molecular mechanisms of spermatogenesis, testicular development, and sperm protein of *H. discus hannai* are not well-known yet. To study the molecular mechanism of sperm protein in Pacific abalone, we have recently reported an axonemal protein 66.0 gene related to sperm motility (Sukhan et al., 2020). In this study, another sperm motility-related gene, Tektin-4, was isolated and characterized from the testis of Pacific abalone. Furthermore, mRNA expression levels of Tektin-4 were studied in several sperm experiments and different cryopreserved sperm. To find out the relation to motility, percent sperm motility and ATP levels in the same experimental sperm were also calculated. Finally, *in situ* hybridization was performed to determine the localization of Tektin-4 in the testis of Pacific abalone.

## 2 Materials and Methods

### 2.1 Experimental Animal and Sample Collection

Three-year-old reproductively mature Pacific abalone (*H. discus hannai*) of both sexes with a mean body weight of 114.08 ± 0.59 g and mean shell length of 80.02 ± 0.36 cm were collected from sea cages of Wando-gun, South Korea. These abalones were then transported to the laboratory of molecular physiology in the Department of Fisheries Science, Chonnam National University, South Korea.

### 2.2 Ethics Statement

Abalone experiments were conducted in accordance with guidelines of the Institutional Animal Care and Use Committee of Chonnam National University (CNU IACUC) under a permission number of CNU IACUC-YS-2020-5 and according to the 14th Article of Korean Animal Protection Law of the Korean government. Abalones were cared for in accordance with the Guidelines for Animal Experiments of Chonnam National University.

### 2.3 Tissue Collection for Gene Cloning and *In Situ* Hybridization

All abalones were anesthetized with 5% MgCl_2_ prior to collection of tissue samples. A total of 10 male Pacific abalones were sacrificed, and their testis tissues were collected for cloning and isolation of Tektin-4 gene. The collected testis samples were washed with 0.1 M phosphate-buffered saline (PBS), immediately snap frozen in liquid nitrogen, and stored at −80°C until extraction of total RNA. A portion of the testis tissue of each abalone was cut, washed with 0.1 M PBS, and fixed in 4% paraformaldehyde (PFA) for *in situ* hybridization.

### 2.4 Tissue Collection for mRNA Expression Analysis

The tissue samples were collected after anesthetizing abalones with 5% MgCl_2_. All collected tissue samples were washed with 0.1 M PBS, immediately snap frozen in liquid nitrogen, and stored at −80°C until total RNA extraction.

#### 2.4.1 Different Organ Tissues of Pacific Abalone

A total of 10 Pacific abalones of both sexes were sacrificed to collect tissue samples of different organs. The collected tissues were cerebral ganglion (CG), pleuropedal ganglion (PPG), ovary (OV), testis (TE), hemocyte (HCY), heart (HRT), cephalic tentacle (CT), epipodial tentacle (ET), gill (GIL), digestive gland (DG), mantle (MA), and muscle (MUS).

#### 2.4.2 Gonads of Abalone at Different Gonadal Developmental Stages

The testis and ovary tissues of Pacific abalones at different gonadal developmental stages were also collected. The gonadal developmental stages of Pacific abalones included the degenerative stage (DS), active stage (AS), ripening stage (RS), and spent stage (SS), as previously described (Sukhan et al., 2020).

#### 2.4.3 Testis of Abalones Exposed to Different Effective Accumulative Temperature

Three-year-old healthy mature male abalones at the gonadal recovery phase were collected from sea cages of abalone aquaculture farms and transported to Tou-Jeong Soosan abalone hatchery in Dolsan-eup, Yeosu-si, Jeollanam-do, South Korea. The collected abalones were reared in cemented tanks with running seawater for conditioning. For early gonadal maturation of abalones, broodstock conditioning was provided at an effective accumulative temperature (EAT) at 18°C for about 4 months to reach EAT 1,500°C-days, as described previously (Sukhan et al., 2021b). After 10 days of conditioning in natural water temperature (around 7°C) in the month of December, abalones were transferred to an EAT-maintained rearing tank. The water temperature was then raised gradually from 7°C to 18°C and maintained at 18°C until the end of the experiment. The abalone samples were sacrificed at 0°C-days (at the start of the experiment), 500°C-days, 1,000°C-days, and 1,500°C-days. At each sampling day, 10 male abalones were sacrificed and the testis tissues were collected from each abalone.

#### 2.4.4 Sperm of Abalones at Peak Breeding Season

The sperm samples of peak breeding season abalones were collected by striping for 4 months in two peak breeding seasons of a year, 2 months during each peak breeding season. Concerning the breeding seasons of abalones, May and June were selected as the first peak breeding season and September and October were selected as the second peak breeding season, as described previously (Cho, 2021).

#### 2.4.5 Sperm of Abalones at Different Steps of Induced Spawning Activity

The sperm samples were collected from abalones at different steps of induced spawning activity such as heat induction (HI), induction with UV-irradiated water (UV), during spawning (DS), and after spawning (AS). Spawning of abalone was induced using fully mature abalones by heating and the UV irradiation method (Leighton, 2008; Sukhan et al., 2021b). Briefly, fully mature abalones were exposed to sunlight, 1 h in upside-down orientation and half an hour in upside-up orientation. The abalones were then induced with UV-irradiated seawater by keeping them in a spawning tank with moderate aeration until spawning. At each induced spawning activity step, sperms were collected from 10 abalones by stripping. Finally, 1 h after spawning, the samples were collected as after spawning samples.

#### 2.4.6 Sperm Cryopreserved in Different Cryoprotectant

To observe mRNA expression of *Hdh-TEKT4* in cryopreserved sperm, sperms were collected from 10 fully mature male Pacific abalones by stripping (Hossen et al., 2021a). Sperm cryopreservation was performed using five types of cryoprotectant solutions, namely, 8% dimethyl sulfoxide (8% DMSO), 8% ethylene glycol (8% EG), 6% propylene glycol (6% PG), 2% glycerol (2% GLY), and 2% methanol (2% MeOH), as reported previously (Kim et al., 2020). Briefly, the sperm samples were diluted with filtered seawater at a ratio of 1:10 (v:v), and the diluted sperm were then mixed with each cryoprotectant solution at a concentration of 1:1 (v:v). The final sperm solutions were equilibrated for 10 min, decanted into 0.50 ml straws, and sealed with straw powder. The straws were then placed in 5 cm rack heights above the liquid nitrogen for 10 min. These straws were then immediately submerged into liquid nitrogen for at least 2 h and finally stored in a liquid nitrogen tank for further analysis.

### 2.5 RNA Extraction and cDNA Synthesis

Total cellular RNAs from all sampled tissues were extracted using an ISOSPIN Cell & Tissue RNA kit (Nippon Gene, Tokyo, Japan). First-strand cDNAs were synthesized from 1∼4 μl of total RNAs using an oligo(dT) primer (Sigma) and superscript III First-strand cDNA synthesis kit (Invitrogen, USA). Using a SMARTer® RACE 5′/3′ Kit (Takara Bio Inc., Japan), 5′- and 3′-RACE cDNAs were synthesized from 1 μl of testis total RNA. All steps of RNA extraction and cDNA synthesis were conducted following the manufacturer’s protocol.

### 2.6 Cloning and Sequencing of Full-Length Tektin-4 Gene (*Hdh-TEKT4*) *in H. discus hannai*

#### 2.6.1 Cloning of Partial Sequence

To obtain a partial fragment of *Hdh-TEKT4* gene, reverse transcription polymerase chain reaction (RT-PCR) was performed using testis cDNA template, a set of forward and reverse primers, and Phusion® High-Fidelity DNA Polymerase (Biolabs Inc., New England). The primer set was designed from a known tektin-A1 nucleotide sequence of *Haliotis asinina* (GenBank Accession no. EU827257.1). All primers used in this experiment are presented in Table 1. The RT-PCR reaction mixture was prepared with a total volume of 20 μl containing cDNA template (1 μl), 20 pmol forward (TEKT4 Fw) and reverse (TEKT4 Rv) primer (1 μl each), HF buffer (4 μl), dNTP mix (2 μl), DNA polymerase (0.5 μl), and sterile distilled water (10.5 μl). The RT-PCR thermal cycling condition was initial denaturation at 95°C for 2 min, followed by 35 cycles of denaturation at 95°C for 30 s, annealing at 58°C for 45 s, extension at 72°C for 30 s, and a final extension step at 72°C for 5 min. The obtained PCR products were subjected to 1.2% agarose gel electrophoresis and purified using a Wizard® SV Gel and PCR Clean-Up System kit (Promega). The purified DNA was ligated into pTOP Blunt V2 vector (Enzynomics, Korea) and transformed into *Escherichia coli* DH5α competent cells (Enzynomics, Korea). The plasmid DNA of positive clones was then purified using a Hybrid-QTM Plasmid Rapidprep mini kit (GeneAll, Seoul, Korea) and sequenced at Macrogen (Seoul, Korea).

**TABLE 1:** List of primers used for cDNA cloning, tissue distribution, and expression analysis of *Hdh-TEKT4* in Pacific abalone.

Primer name	Nucleotide sequence (5′--- 3′)	Purpose
TEKT4—Fw	TGT​CAT​GAG​GTC​AGA​ATC​AGG	RT-PCR
TEKT4—Rv	CTG​AGC​AAG​CAA​CAA​GTC​TG	
Hdh-TEKT4—3′RACE	GAT​TAC​GCC​AAG​CTT​ACA​CAG​TCC​GAG​GTG​ACC​AAG​AAG​CTG	RACE PCR
Hdh-TEKT4—5′RACE	GAT​TAC​GCC​AAG​CTT​CAG​TGT​TGC​CAG​TGT​AAA​CCG​TCT​CCA​T	
Universal primer (short)	CTA​ATA​CGA​CTC​ACT​ATA​GGG​C	
Universal primer (long)	CTA​ATA​CGA​CTC​ACT​ATA​GGG​CAA​GCA​GTG​GTA​TCA​ACG​CAG​AGT	
Hdh-TEKT4—Fw	TCC​GAG​GTG​ACC​AAG​AAG​C	qRT-PCR
Hdh-TEKT4—Rv	CAG​TTC​AGA​TTG​TCT​GTT​GCA	
Hdh-β-actin—Fw	CCG​TGA​AAA​GAT​GAC​CCA​GA	
Hdh-β-actin—Rv	TAC​GAC​CGG​AAG​CGT​ACA​GA	
Hdh-TEKT4—sense	AAG​TTG​CGC​CAC​GAA​TCA​GAG	ISH
Hdh-TEKT4—antisense	CAA​CCT​TGA​GTG​GGT​CTT​CCT​T	
Oligo dT (OdT)	GGC​CAC​GCG​TCG​ACT​AGT​ACT​TTT​TTT​TTT​TTT​TTT​T	cDNA synthesis
Oligo dT adapter (AP)	GGC​CAC​GCG​TCG​ACT​AGT​AC	

#### 2.6.2 Cloning of 5′- and 3′-RACE Sequence

A SMARTer® RACE 5′/3′ kit (Takara Bio Inc., Japan) was used to perform 5′- and 3′-rapid amplification of cDNA ends (RACE) to obtain the full-length sequence of *Hdh-TEKT4*. A set of gene-specific 5′- and 3′-RACE primers was prepared from the obtained partial sequence including a 15-bp (GATTACGCCAAGCTT) overlap at the 5′-end of the primer sequence. 5′- and 3′-RACE PCRs were executed using 2.5 μl of 3′- or 5′-RACE cDNA, 1 μl of sense (Hdh-TEKT4-3′RACE) or antisense (Hdh-TEKT4-5′RACE) RACE primer, 5 μl of a universal primer mix (UPM), 1 μl of SeqAmp DNA polymerase, 25 μl of SeqAmp buffer, and 15.5 μl of PCR-grade water. Touchdown PCR was carried out for 30 cycles for both 3′-RACE and 5′-RACE. The thermal cycle conditions were maintained as prescribed in the kit. RACE PCR products were subjected to 1.2% agarose gel electrophoresis and purified using a NucleoSpin® Gel and PCR Clean-up kit (MACHEREY-NAGEL GmbH & Co. KG, Germany). The purified RACE PCR products were ligated into linearized pRACE vector, transformed into Stellar competent cells, and sequenced as described previously for the partial sequence. Finally, the sequences of RACE products were combined, and overlaps with the initially cloned partial cDNA fragment were trimmed to obtain the full-length sequence.

### 2.7 Sequence Analysis of Cloned *H. discus hannai* Tektin-4 Sequence

A number of online tools were used to analyze nucleotide and amino acid sequences of the cloned *Hdh-TEKT4*. The deduced amino acid sequence of *Hdh-TEKT4* cDNA was generated from the cloned full-length nucleotide sequence using the EMBOSS Transeq (http://www.ebi.ac.uk/Tools/st/emboss_transeq/) online tool. Open reading frames (ORFs) and potential protein encoding segments were predicted from the nucleotide sequence using ORFfinder (https://www.ncbi.nlm.nih.gov/orffinder/). The protein homology of Hdh-TEKT4 was analyzed using the Basic Local Alignment Search Tool (BLASTP; http://www.ncbi.nlm.nih.gov/BLAST/). The molecular weight and theoretical isoelectric point (pI) of the protein were computed using ProtParam (https://web.expasy.org/protparam/) and Protcomp 9.0 (http://www.softberry.com/berry.phtml) online tools, respectively. The gene ontology of Hdh-TEKT4 protein was predicted using an online tool, Contact-guided Iterative Threading ASSEmbly Refinement (C-I-TASSER) protein structure prediction server (https://zhanggroup.org/C-I-TASSER/; Zheng et al., 2021). The functional domains of the protein were determined using NCBI conserved domain search program (http://www.ncbi.nlm.nih.gov/Structure/cdd/wrpsb.cgi), Motif scan (http://myhits.isb-sib.ch/cgi-bin/motif_scan), SMART (http://smart.embl-heidelberg.de/), or InterProScan (http://www.ebi.ac.uk/InterProScan/). The conserved motifs in the Hdh-TEKT4 amino acid sequence were discovered using Multiple Em for Motif Elicitation (MEME) online tools (v. 5.0.5; http://meme-suite.org/tools/meme; Bailey et al., 2006). The coiled–coil region in the protein sequence was predicted using an online program COILS with 28 width, MTIDK matrix, and 2.5 weighting of hydrophobic positions a and d (http://www.ch.embnet.org/software/COILS_form.html). The amino acid sequences of Hdh-TEKT4 and related proteins were aligned using an online based multiple sequence alignment program, ClustalOmega (https://www.ebi.ac.uk/Tools/msa/clustalo/). The alignment of protein sequences was edited and visualized using Jalview version 2.11.1.7 software (Waterhouse et al., 2009).

### 2.8 Phylogenetic Analysis

A phylogenetic tree was constructed using Tektin-4 and its homologous protein sequences from different organisms. The Tektin-4 amino acid sequence of *H. discus hannai* was aligned with related protein sequences using an online tool, ClustalW. The phylogenetic tree was constructed using MEGA software (v. 11) with a neighbor-joining algorithm (Tamura et al., 2021). The numbers shown on the branches indicate the significance of nodes based on a bootstrap analysis conducted on 1,000 replicates.

### 2.9 Homology Modeling of *Hdh-TEKT4*

The three-dimensional (3D) structure of Hdh-TEKT4 was generated using an online protein structure and functional prediction program, I-TASSER (Iterative Threading ASSEmbly Refinement) server (https://zhanglab.ccmb.med.umich.edu/I-TASSER/; Roy et al., 2010). The predicted 3D structure of Hdh-TEKT4 was analyzed and visualized using UCSF ChimeraX software (v. 1.2.5) (https://www.cgl.ucsf.edu/chimera/).

### 2.10 Fluorescence *In Situ* Hybridization (FISH) Localization of *Hdh-TEKT4* in Testis of Pacific Abalone

#### 2.10.1 Riboprobe Synthesis

Riboprobes were synthesized using standard protocol, as described previously (Sukhan et al., 2013), with a few modifications. Anti-sense and sense fluorescence mRNA probes were prepared from 613 bp fragments from the Tektin domain of *Hdh-TEKT4* that was amplified using a set of anti-sense and sense primers (Table 1) and subcloned in pGEM-T easy vector (Promega, USA). The linear plasmid was prepared using 10 µg of *Hdh-TEKT4* cDNA fragments with restriction enzymes SalI or NcoI (Promega, USA). Anti-sense and sense riboprobes were labeled separately with fluorescein-12-UTP (Roche, Germany) using T7 or SP6 RNA polymerases (Promega). Then, 1 microgram of the linearized plasmid DNA was incubated at 37°C for 2 h with 2.0 µl of T7 or SP6 RNA polymerase in a total volume of 20 µl containing 5x optimized transcription buffer (4.0 µl), 100 mM DTT (2.0 µl), fluorescein RNA labeling mix (2.0), RNase inhibitor (2.0), and RNase-free water (8.0 µl). After the transcription reaction, the linearized plasmid DNA template was digested at 37°C for 15 min with RNase out (0.5 µl) and DNase I (2.0 µl). These riboprobes were purified by ethanol precipitation with 1 μl of yeast tRNA (10 mg/ml, Sigma) and stored at −80°C until used for ISH.

#### 2.10.2 Preparation of Frozen Tissue Sections

PFA-fixed Pacific abalone testis tissues were infiltrated in 30% sucrose and embedded in optimum cutting temperature (OCT) compound (FSC 222, Leica Biosystems, Wetzlar, Germany). These testis tissues were then sectioned at 8 µM thickness in transverse orientation using a cryostat device (CM 3050; LEICA, Wetzlar, Germany). The sections were collected onto SuperFrost®Plus slides (VWR International, Radnor, PA, Unites States) and immediately stored at −20°C until further use.

#### 2.10.3 Fluorescence *In Situ* Hybridization

FISH was carried out following the standard protocol (Schütz et al., 2006) and DIG *in situ* hybridization application manual with slight modification. Briefly, a hybridization buffer was prepared in a total volume of 50 ml using 25 ml of deionized formamide, 12.5 ml of 20× saline sodium citrate (SSC), 0.5 ml of 0.1% Tween-20, 0.46 ml of 1M citric acid (pH 6.0), and DEPC-H_2_O. The cryosections of testis tissue were first prehybridized with hybridization buffer mix with yeast tRNA (9:1 ratio) at 65°C for 2 h, followed by hybridization with fluorescein-12-UTP-labeled RNA probe (200 ng/ml, diluted with hybridization buffer mix) at 65°C for overnight. The tissue sections were then subsequently washed with degraded series (75%, 50%, 25% volume) of hybridization mix with 2× SSC for 10 min each at 65°C. Next, the sections were washed with 2× SSC and 0.2× SSC for 15 min each. After that, the sections were sequentially washed with degraded series of 0.2× SSC (75%, 50%, 25% volume) mixed with PBST and then with PBST for 5 min each at room temperature. To detect the hybridization signal, the tissue sections were then incubated with 10% calf serum at room temperature for 1 h and then incorporated with anti-digoxigenin-fluorescein, Fab fragments antibody (diluted 1:500 in 10% calf serum) at room temperature for 1 h. The tissue sections were then washed with PBST three times for 10 min each, followed by washing three times with alkaline-Tris buffer for 5 min each at room temperature. Finally, the sections were counterstained and mounted using VECTASHIELD antifade mounting medium with DAPI (4,6′-diamidino-2-phenylindole) (Vector Laboratories, Inc., USA). The fluorescence signals were visualized and captured using a ZEISS LSM 900 with Airyscan2 confocal microscope (ZEISS, Germany). The images were processed with ZEISS ZEN 3.2 (Blue edition) software.

### 2.11 Semiquantitative Reverse Transcription Polymerase Chain Reaction

Semiquantitative RT-PCR was performed using gene-specific forward and reverse primers to observe the tissue distribution pattern of *Hdh-TEKT4* gene in various tissues of Pacific abalone. The gene expression levels were observed in 12 different tissues (CG, PPG, OV, TE, HCY, HRT, CT, ET, GIL, DG, MNT, and MUS) and in tissues at different gonadal developmental stages (DS, AS, RS, and SS) of both sexes of Pacific abalone. *H. discus hannai β-actin* (GenBank accession no. AY380809) was used as an internal control due to its expression stability. RT-PCR was performed with a 20 µl reaction mixture containing cDNA template (1 µl), forward and reverse primer (1 µl each), 2x Prime Taq premix (10 µl) (GENETBIO, Korea), and sterilized distilled water (7 µl). The PCR thermal cycling conditions were the same as those used for partial sequencing.

### 2.12 Quantitative Real-Time PCR Analysis

To quantify relative mRNA abundance of *Hdh-TEKT4* in different tissues, qRT-PCR analysis was performed. *Hdh-TEKT4* mRNA expression levels were observed in 12 different tissues (CG, PPG, OV, TE, HCY, HRT, CT, PT, GIL, DG, MA, and MUS), testis tissues at different testicular developmental stages (DS, AS, RS, and SS) of abalones, testis tissues at different EAT °C-days (0, 500, 1,000 and 1,500°C-days) maintained under effective accumulative temperature exposure for early gonadal maturation, sperm of peak breeding season (May, June, September, October) abalone, sperm collected at different steps during induced spawning activity, and sperm cryopreserved in different cryoprotectant.

The qRT-PCR assay was conducted using a 2×qPCRBIO SyGreen Mix Lo-Rox kit (PCR Biosystems Ltd., UK), as described previously (Kho et al., 2021). The qRT-PCR reaction mixture was prepared with cDNA template (1 μl), 10 pmol forward and reverse primer (1 μl each), SyGreen Mix (10 μl), and ultra-pure water (10 μl) in a total volume of 20 μl. Triplicate reactions were performed for target and reference genes in each tissue sample. The PCR amplification conditions were preincubation at 95°C for 2 min, followed by 40 cycles of a three-step amplification at 95°C for 30 min, 60°C for 20 s, and 72°C for 30 s. The melting temperature was used as the instrument’s default setting. At the end of each cycle, a fluorescence reading was recorded for quantification. A LightCycler® 96 System (Roche, Germany) was used for amplification and data analysis. The relative gene expression was determined using the 2^−ΔΔCT^ method (Livak and Schmittgen 2001) with *H. discus hannai β-actin* gene as an internal reference.

### 2.13 Counting of Sperm Motility

Sperm motility was determined for cryopreserved sperm, sperm at peak breeding season, and sperm at different spawning activity steps, as described previously (Hossen et al., 2021b). A small volume of sperm was diluted at a 1:10 ratio using filtered seawater in a micro tube. Later, 2 µl of diluted sperm was added to 98 µl filtered seawater on a glass slide to observe and count the number of motile sperm. The motility of each replication was calculated based on the average value of ten sub-samples as % motility.

### 2.14 Measurement of Sperm Adenosine Triphosphate Levels

The sperm ATP levels were measured for peak season fresh sperm and cryopreserved sperm using an ATP assay kit (BIOMAX, Seoul, Korea) according to the manufacturer’s instructions. Briefly, the collected sperm were homogenized in a 100 μl assay buffer and later resuspended in an assay buffer to a concentration of 1 × 10^6^ cells/ml. Then, 50 μl of each sample was transferred to a well in a 96-well plate. Subsequently, a 50 μl ATP reaction mixture was added to each well and mixed by gentle shaking for 2 min in a rotary shaker to induce cell lysis. After 30 min of incubation at room temperature in the dark, the absorbance was detected at 570 nm using a microplate reader (Epoch 2; BioTek Instruments, Inc., USA). To generate a standard curve, ATP standard solutions were prepared from 10 mM ATP standard. The absorbance value for each sample was converted to the corresponding ATP concentrations (nM) using the standard curve.

### 2.15 Statistical Analysis

Values of mRNA expression, sperm motility, and ATP levels were analyzed statistically and expressed as mean ± standard error of the mean (SEM). The changes in relative mRNA expression, sperm motility, and ATP levels in different testis and sperm samples were analyzed by nonparametric one-way analysis of variance (ANOVA) using GraphPad Prism 9.3.1 software. Tukey’s post hoc test was performed to assess statistically significant differences among different experimental tissues. The statistical significance was set at *p* < 0.05. All graphs were prepared using GraphPad Prism 9.3.1 software. The different letters on the bar in figures and in tables indicate significant differences.

## 3 Results

### 3.1 *Haliotis discus hannai* Tektin-4 Sequence

**FIGURE 1:**
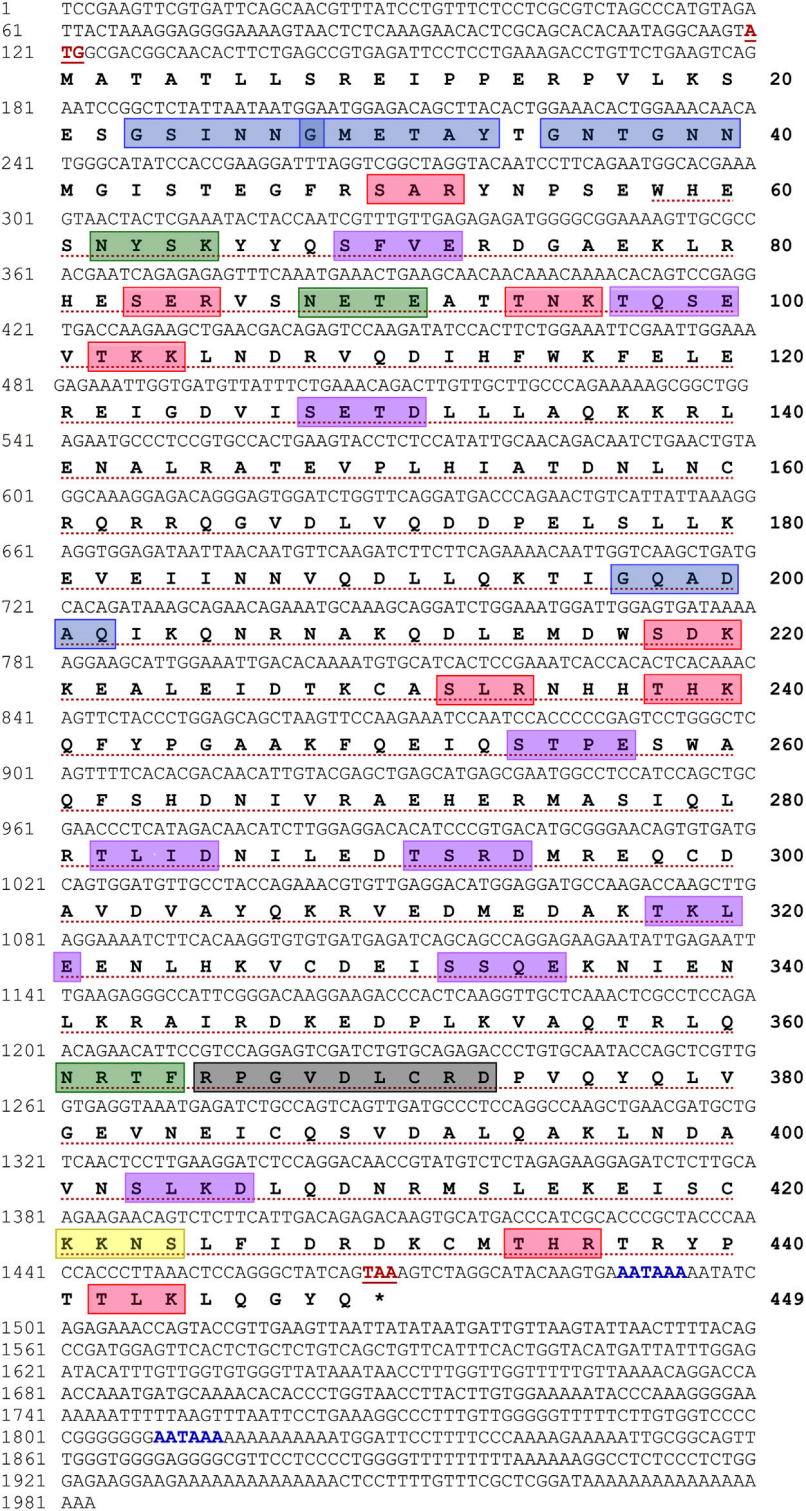
Full-length nucleotide and deduced amino acid sequences of *Hdh-TEKT4* (GenBank accession no. MZ265399). Numbers at the left and right sides indicate nucleotide and amino acid positions in the sequence, respectively. Start and stop codons are shown in bold red and underlined. Putative polyadenylations signals (AATAAA) are shown in bold blue. The tektin domain is marked with red dotted underline. A variant of conserved tektin nonapeptide signature sequence is indicated in a light black box. Predicted protein kinase C (PKC) phosphorylation sites are boxed in red. Potential casein kinase II phosphorylation sites are boxed in purple. cAMP- and cGMP-dependent protein kinase phosphorylation site is boxed in yellow. Potential N-linked glycosylation sites are boxed in green, and N-myristoylation sites are boxed in blue.

A cDNA sequence encoding *H. discus hannai Tektin-4* (*Hdh-TEKT4*) was cloned and sequenced from the testis tissue of Pacific abalone (Figure 1). The full-length sequence of *Hdh-TEKT4* cDNA (GenBank accession no. MZ265399) was 1,983 bp long including a poly-A tail. Its 5′- and 3′-untranslated regions (UTR) were 119 bp and 514 bp long, respectively. Two putative polyadenylation signals (AATAAA) were found in its nucleotide sequence at 151 and 471 bp upstream of poly-A tail. The open reading frame (ORF) of the *Hdh-TEKT4* cDNA sequence was 1,350 bp, encoding a putative protein with 449 deduced amino acids.

### 3.2 Features of Hdh-TEKT4 Amino Acid Sequence and Bioinformatics Analysis

The theoretical molecular weight and isoelectric point (pI) of the Hdh-TEKT4 protein were 51.83 kDa and 5.50, respectively. The aliphatic index of the protein was 75.81. The instability index (II) was computed as 48.74, indicating it as an unstable protein. The neural nets nuclear prediction and integral prediction of protein location scores were 1.8 and 4.0, respectively, which predicted the protein as cytoplasmic protein. Gene ontology (GO) term analysis using C-I-TASSER server predicted Hdh-TEKT4 protein as the movement of cell or subcellular component protein (GO: 0006928) in biological processes (Supplementary Figure S1A) with a C-score^GO^ of 0.68, cytoskeleton protein (GO:0005856) in cellular component with a C-score^GO^ of 0.56 (Supplementary Figure S1B), and cytoskeletal binding protein (GO:0008092) in molecular function with a C-score^GO^ of 0.53 (Supplementary Figure S1C).

Motif scan and conserved domain search suggested that Hdh-TEKT4 had a Tektin domain at a position of 58–440 amino acid residues with an E-value of 2.1e-152. This protein also had a variant of nonapeptide signature sequence (RPGVDLCRD) at 365–373 amino acids, which is conserved in all the members of the tektin family. The phosphorylation site search revealed that it had nine protein kinase C (PKC) phosphorylation sites, [S/T]-X-[R/K], at positions 50–52 (SAR), 83–85 (SER), 94–96 (TNK), 102–104 (TKK), 218–220 (SDK), 232–234 (SLR), 238–240 (THK), 434–436 (THR), and 442–444 (TLK); nine casein kinase II phosphorylation sites, [S/T]-X(2)-[D/E], 69–72 (SFVE), 97–100 (TQSE), 128–131 (SETD), 254–257 (STPE), 282–285 (TLID), 291–294 (TSRD), 318–321 (TKLE), 332–335 (SSQE), and 403–406, (SLKD); and one cAMP- and cGMP-dependent protein kinase phosphorylation site, [R/K](2)-X-[S/T], at position 421–424 (KKNS). Three N-linked glycosylation sites, N-{P}-[S/T]-{P}, were found at positions 62–65 (NYSK), 88–91 (NETE), and 361–364 (NRTF). Four N-myristoylation sites, G-{EDRKHPFYW}-X(2)-[S/T/A/G/C/N]-{P}, were found at positions 23–28 (GSINNG), 28–33 (GMETAY), 35–40 (GNTGNN), and 197–202 (GQADAQ) of the deduced Hdh-TEKT4 protein.

**FIGURE 2:**
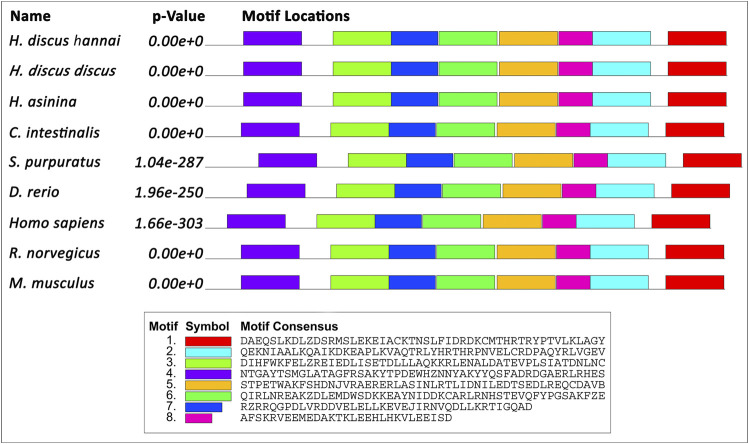
Schematic diagram of the motif detected in Hdh-TEKT4 and other TEKT4 of invertebrates and vertebrates. Distinct motifs are denoted by different colors. The motif analysis included Hdh-TEKT4 amino acid sequence and various other sequences of TEKT4: Tektin-4 of *H. discus discus* (ARR97156), *Homo sapiens* (NP_653306), *Danio rerio* (NP_001139162), *Rattus norvegicus* (BAD93477), *Mus musculus* (NP_082227), tektin-A1 identical to tektin-4 of *H. asinina* (ACJ15284), *Ciona intestinalis* (NP_001027644), and *Strongylocentrotus purpuratus* (NP_999787).

Motifs of Tektin-4 were analogously expressed when they were compared with different Tektin-4 protein sequences with motif width between 24 and 50 amino acids. A total of eight motifs were recognized in Hdh-TEKT4. Similarly, eight motifs were recognized in other compared Tektin-4 proteins (Figure 2).

**FIGURE 3:**
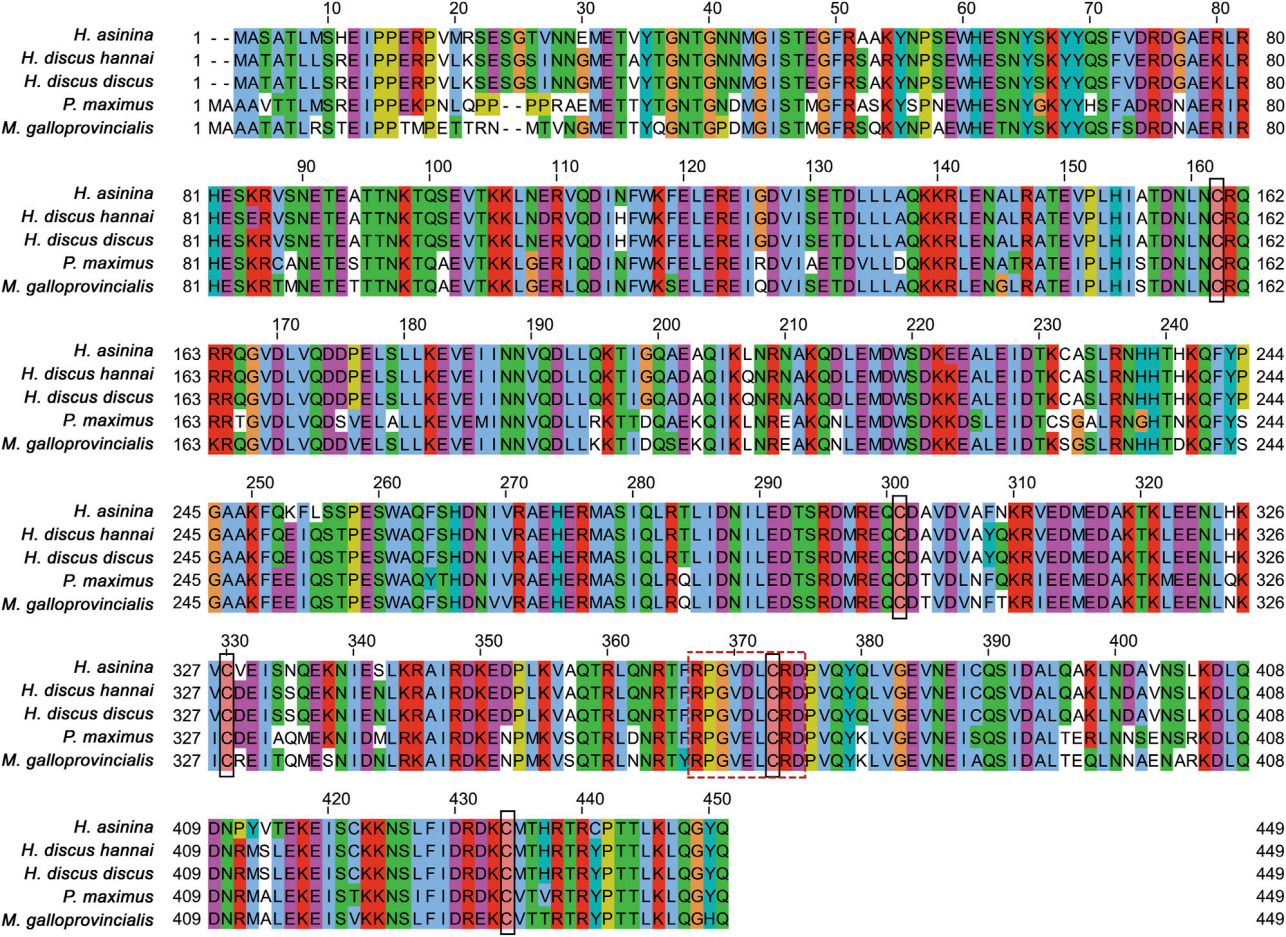
Multiple sequence alignment of deduced amino acid sequences of different tektins from *H. discus hannai* (MZ265399.1), *H. discus discus* (ARR97156.1), *H. asinina* (ACJ15284.1), *Pecten maximus* (XP_033751776.1), and *Mytilus galloprovincialis* (VDI53310.1). Dashes indicate gaps. Five cysteine residues conserved in the sequence alignment are shown in the black lined box. Variants of tektin nonapeptide signature sequences are boxed with a break line.

Multiple sequence alignment revealed that five cysteine residues at positions 160, 299, 328, 371, and 432 of the Hdh-TEKT4 deduced amino acid sequence were conserved when aligned with Tektin-4 and related proteins of *H. discus hannai*, *H. discus discus*, *H. asinina*, *P. maximus*, and *M. galloprovincialis* (Figure 3). Among compared invertebrate Tektin-4-related protein sequences, Hdh-TEKT4 showed the highest identity and similarity to Tektin-4-like protein of *H. discus discus* (at 97.59% and 89%, respectively). It also showed 89.88% identity and 86% similarity to Tektin A1 of *H. asinina*.

### 3.3 Structure of the Hdh-TEKT4 Protein

**FIGURE 4:**
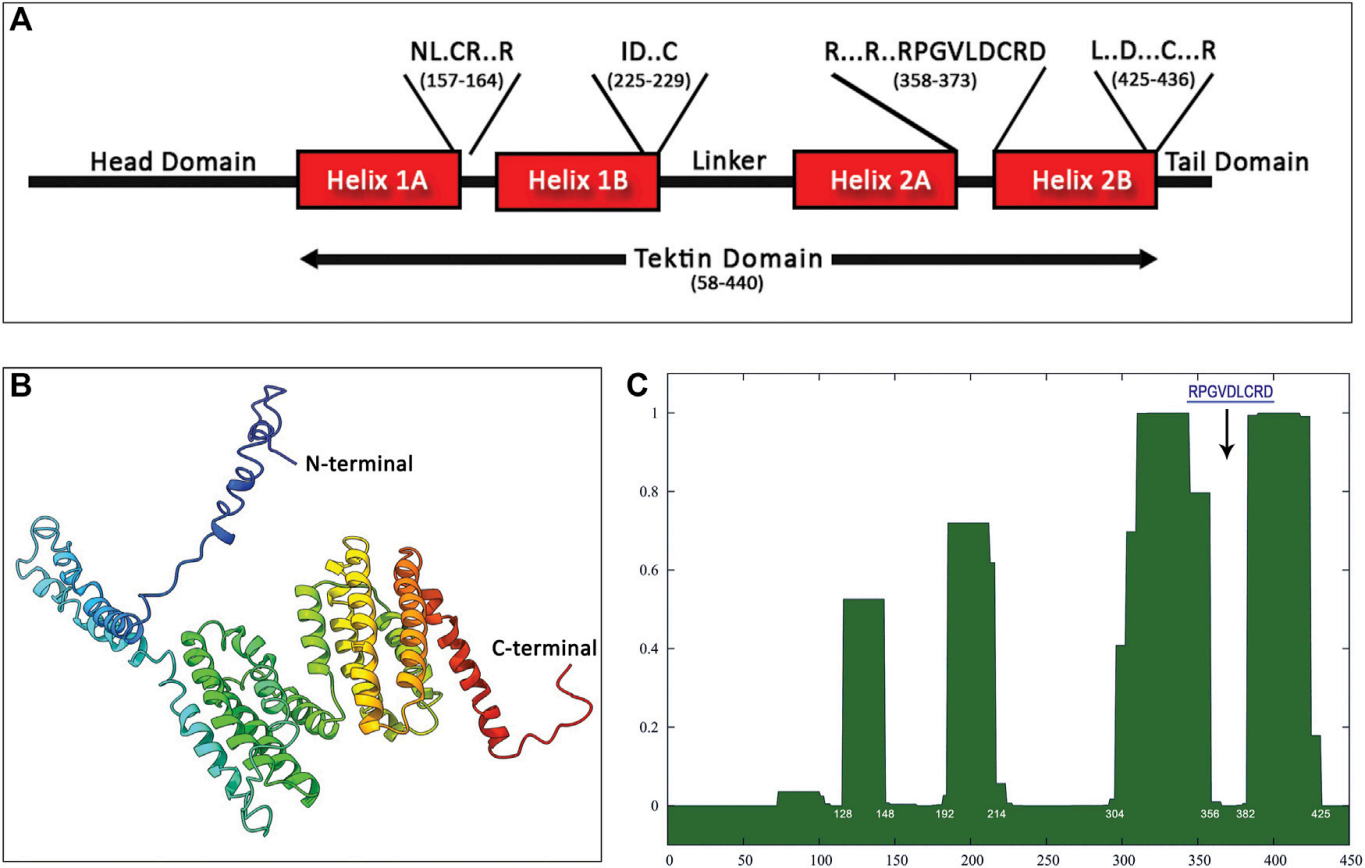
Structure prediction of Hdh-TEKT4 from amino acid sequence. **(A)** Apparent domain structure within Hdh-Tektin-4. Positions of some conserved motifs reported by Amos (2008), including the signature nonapeptide, are indicated in a single-letter amino acid code in the diagram with the amino acid position. **(B)** Three-dimensional homology modeling of Tektin-4 of *Haliotis discus hannai*. The model was constructed using I-TASSER online tools. Domains between the N terminus and C terminus were predicted from the secondary structure. **(C)** Predictions of coiled–coil segments from amino acid sequences of Hdh-TEKT4. The x-axis represents the amino acid number, and the y-axis represents the probability (0.0–1.0) that the sequence will form a coiled–coil secondary structure. The position corresponds to that of the tektin loop containing the conserved nonapeptide shown by a down-arrow.

The Hdh-TEKT4 protein consisted of amino- and carboxy-terminal head and tail domain on each side of a conserved coiled–coil tektin domain (Figure 4A). The tektin domain (54-440) was divided in halves and each half was further divided into two. Thus, the Hdh-TEKT4 protein might be equivalent to a quarter. These four α-helical segments, helix 1A and helix 1B at the N terminus and helix 2A and helix 2B at the C terminus, were connected by linkers. The tektin signature nonapeptide sequence (RPGVDLCRD) appeared in the middle of the second half, in the linker between 2A and 2B helices. Furthermore, two highly conserved NL×CR××R and R×××R××RPGVLDCRD motifs were found between helix 1A and helix 1B, and helix 1B and helix 2A, respectively. Two additional conserved motifs, ID××C, L××D×××C×××R, were found at the C-terminal end of helix 1B and helix 2B, respectively.

The three-dimensional structure of the Hdh-TEKT4 protein showed multiple alpha helices separated by a helix–loop–helix structure (Figure 4B). The protein sequence analysis of Hdh-TEKT4 using SMART predicted four coiled–coil domains located at residues of 128–148, 192–214, 304–356, and 382–425 amino acids. The analysis using the online program COILS revealed the same four coiled–coil domains in the Hdh-TEKT4 protein (Figure 4C). The existence of coiled–coil domains revealed that this protein is consistent with the characteristic of a filament-forming tektin protein.

### 3.4 Phylogenetic Analysis

**FIGURE 5:**
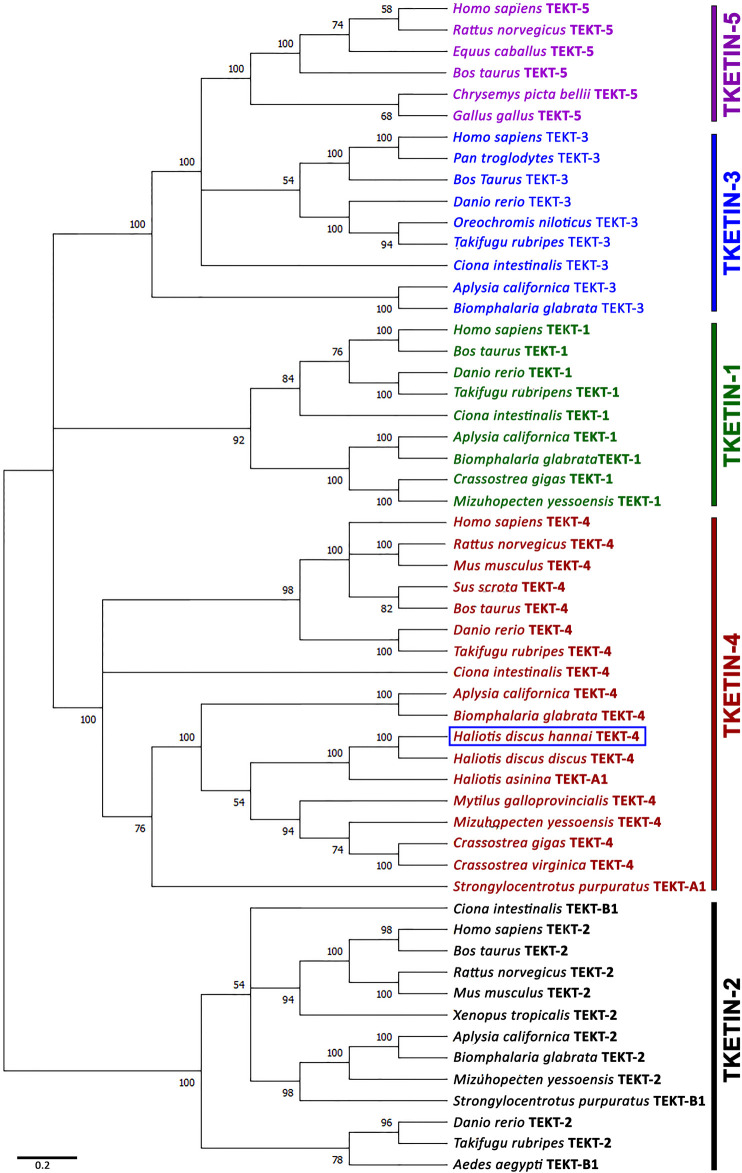
Phylogenetic tree constructed by the bootstrap neighbor-joining method after clustalW alignment based on amino acid residues of different isoforms of tektins. Numbers at the nodes indicate bootstrap probability. GenBank accession numbers of sequences used to construct the phylogenetic tree are as follows: Tektin-1 of *H. sapiens* (NP_444515.1), *Bos taurus* (NP_001069251.1), *D. rerio* (XP_009290074.1), *Takifugu rubripes* (XP_011607045.1), *C. intestinalis* (XP_002130466.1), *Aplysia californica* (XP_005109632.1), *Biomphalaria glabrata* (XP_013064715.1), *Crassostrea gigas* (XP_011416301.1), *Mizuhopecten yessoensis* (OWF41001.1); tektin-2 or tektin-B1 of *H. sapiens* (NP_055281.2), *B. taurus* (NP_001033192.1), *Rattus norvegicus* (NP_001011977.1), *M. musculus* (NP_036032.2), *Xenopus tropicalis* (NP_001007884.1), *D. rerio* (NP_001017432.3), *T. rubripes* (XP_003969284.2), *Aedes aegypti* (XP_001657769.1), *C. intestinalis* (NP_001027645.1), *A. californica* (XP_005098539.1), *B. glabrata* (XP_013074221.1), *M. yessoensis* (OWF46183.1), *S. purpuratus* (NP_999789.1); tektin-3 of *H. sapiens* (NP_114104.1), *Pan troglodytes* (NP_001233472.1), *B. taurus* (NP_001092489.1), *D. rerio* (XP_701169.2), *Oreochromis niloticus* (XP_003453437.1), *T. rubripes* (XP_003961064.1), *C. intestinalis* (XP_002129626.1), *A. californica* (XP_012939623.1), *B. glabrata* (XP_013095021.1); tektin-4 or tektin-A1 of *H. sapiens* (NP_653306.1), *R. norvegicus* (BAD93477.1), *M. musculus* (NP_082227.1), *Sus scrofa* (AFM37359.1), *B. taurus* (NP_001033158.1), *D. rerio* (NP_001139162.1), *T. rubripes* (XP_003972094.2), *C. intestinalis* (NP_001027644.1), *A. californica* (XP_005092496.1), *B. glabrata* (XP_013090287.1), *H. discus hannai* (MZ265399.1), *H. discus discus* (ARR97156.1), *H. asinina* (ACJ15284.1), *M. galloprovincialis* (VDI53310.1), *M. yessoensis* (OWF46480.1), *C. gigas* (XP_011416098.2), *C. virginica* (XP_022318607.1), *S. purpuratus* (NP_999787.1); tektin-5 of *H. sapiens* (NP_653275.1), *R. norvegicus* (NP_001014246.1), *Equus caballus* (XP_023472090.1), *Chrysemys picta bellii* (XP_005306767.1), and *Gallus gallus* (XP_414934.3). The scale bar indicates 0.2 units of the expected fraction of amino acid substitutions (1.0 unit = 100 PAMs).

A phylogenetic tree was constructed using the neighbor-joining method to assess Hdh-TEKT4 and its possible evolutionary connections to other tektin proteins. An unrooted phylogenetic tree based on the amino acid sequences of Tektin proteins from various species showed five major groups: Tektin-1, Tektin-2, Tektin-3, Tektin-4, and Tektin-5 (Figure 5). Hdh-TEKT4 was fitted with the Tektin-4 group and clustered with its phylogenetically closest matches, Tektin-4 of *H. discus discus* and Tektin A1 of *H. asinina*.

### 3.5 *In Situ* Hybridization Localization of *Hdh-TEKT4* in the Testis of Pacific Abalone

**FIGURE 6:**
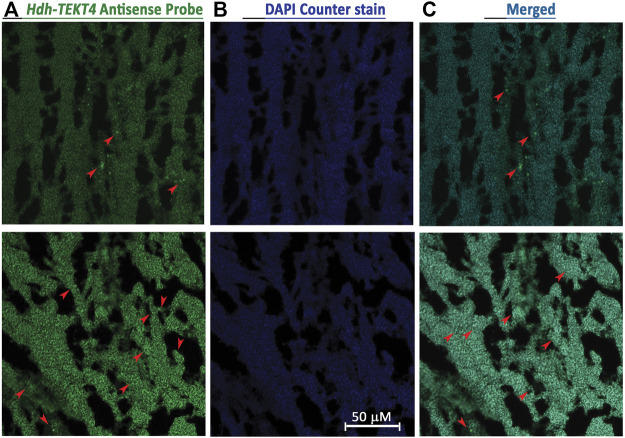
Confocal laser scanning microscopic observation after fluorescence *in situ* hybridization (FISH) of *Hdh-TEKT4* mRNA in fully mature testis tissue of Pacific abalone. **(A)** Single confocal optical sections showing a positive signal of *Hdh-TEKT4* mRNA (green) when hybridized with the anti-sense probe. **(B)** Nuclei are counterstained with DAPI (blue). **(C)** Merged image of **(A,B)**. Scale bar: 50 μm.

Fluorescence *in situ* hybridization was performed to identify cell types expressing *Hdh-TEKT4* mRNA in the testis of Pacific abalone. Confocal laser image scanning showed that hybridization with the antisense probe expressed positive signals in spermatids of Pacific abalone testis (Figure 6). Hybridization with the sense probe of *Hdh-TEKT4* did not show any signal (data not shown). These data indicate that *Hdh-TEKT4* mRNA is exclusively expressed in spermatids of the testis of Pacific abalone.

### 3.6 Semiquantitative PCR Analysis for *Hdh-TEKT4* Tissue Distribution

The tissue distribution analysis revealed that the *Hdh-TEKT4* gene was mainly expressed in testis tissues. It had very weak expression in CT and PT (Supplementary Figure S2). In the case of different gonadal developmental stages, its expression was detected in all testicular developmental stages with a dominant expression in RS. However, no expression of *Hdh-TEKT4* was observed in any ovarian developmental stages (Supplementary Figure S3).

### 3.7 Expression of *Hdh-TEKT4* in Different Tissues of Pacific Abalone

**FIGURE 7:**
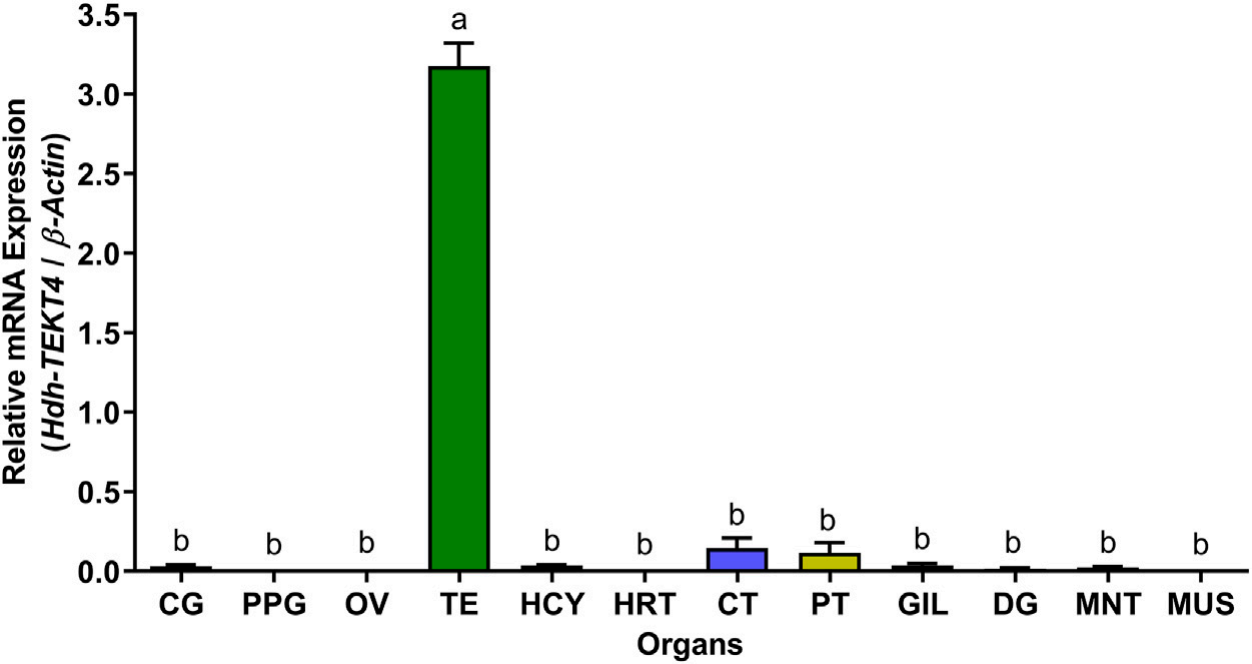
Relative mRNA expression levels (2^–ΔΔCT^) of *Hdh-TEKT4* (mean ± SEM) in different tissues of Pacific abalone detected by qRT-PCR. Different letters above the bars indicated significant differences (*p* < 0.05) among organs. CG, cerebral ganglion; PPG, pleuropedal ganglion; OV, ovary; TE, testis; HCY, hemocyte; HRT, heart; CT, cephalic tentacle; PT, pleuropedal tentacle; GIL, gill; DG, digestive gland; MNT, mantle; MUS, muscle.

The relative mRNA expression levels of *Hdh-TEKT4* in various tissues were analyzed by qRT-PCR, and the results are presented in Figure 7. The *Hdh-TEKT4* mRNA expression level was significantly higher in the testis tissue, followed by that in CT and PT, which showed significantly different expression levels of *Hdh-TEKT4* from other examined tissues. A negligible expression of *Hdh-TEKT4* was observed in CG, PPG, OV, DG, GIL, HRT, HCY, MNT, and MUS tissues.

### 3.8 Expression of *Hdh-TEKT4* in the Testis of Different Testicular Developmental Stages of Pacific Abalone

**FIGURE 8:**
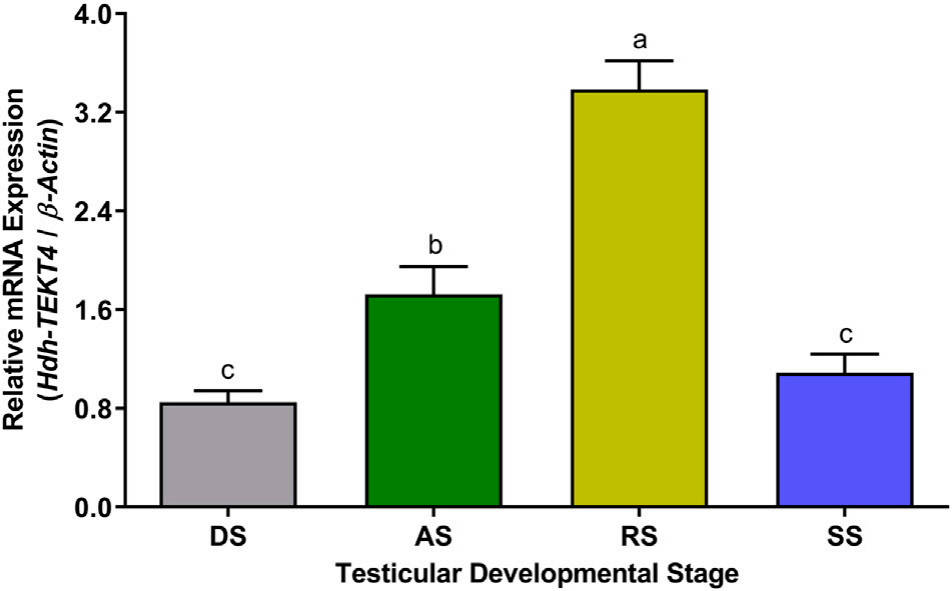
Expression levels of *Hdh-TEKT4* mRNA in the testis of different testicular developmental stages of Pacific abalone. Different letters above the bars indicate significant differences (*p* < 0.05) among developmental stages. DS, degenerative stage; AS, active stage; RS, ripening stage; SS, spent stage.

In different testicular developmental stages, changes in relative mRNA expression of *Hdh-TEKT4* were observed among different stages. A significantly higher expression of *Hdh-TEKT4* was observed in the RS (Figure 8). The relative abundance of *Hhd-TEKT4* mRNA transcript was markedly increased from DS to RS but decreased in SS.

### 3.9 Expression of *Hdh-TEKT4* in Testis of EAT Exposed Pacific Abalone During Broodstock Conditioning

**FIGURE 9:**
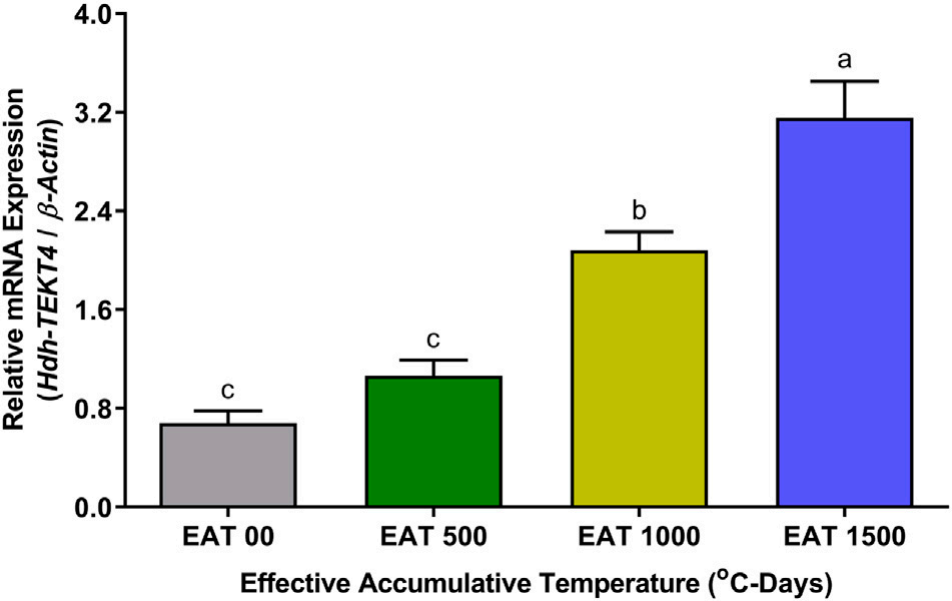
Expression levels of *Hdh-TEKT4* mRNA in the testis of different °C-days of Pacific abalone exposed to effective accumulative temperature during broodstock conditioning. Different letters above the bars indicate significant differences (*p* < 0.05) among °C-days. EAT 00, EAT 0°C-days; EAT 500, EAT 500°C-days; EAT 1000, EAT 1000°C-days; EAT 1500, EAT °C-days.

The expression levels of *Hdh-TEKT4* mRNA in the testis of EAT exposed Pacific abalone during the broodstock condition were found to be significantly higher at EAT 1,500°C-days when abalones were fully mature and ready to spawn (Figure 9). The mRNA expression of *Hdh-TEKT4* was significantly increased from EAT 500°C-days to EAT 1000 1,500°C-day to EAT 1,500°C-day with the gradual progress of maturation of testis. Insignificant changes of mRNA expression of *Hdh-TEKT4* were observed between EAT 0°C-days and EAT 500°C-days when the testis was in an inactive stage.

### 3.10 Expression of *Hdh-TEKT4* mRNA and Percent Motility in Sperm of Peak Breeding Season of Pacific Abalone

**FIGURE 10:**
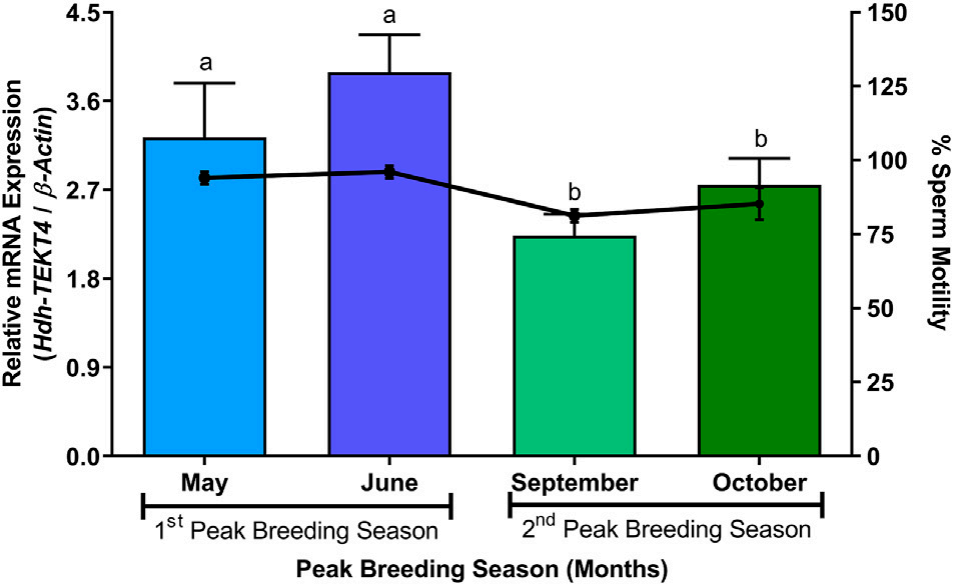
Expression levels of *Hdh-TEKT4* mRNA and percent sperm motility in sperm of Pacific abalone during different months of peak breeding seasons. Different letters above the bars indicate significant differences (*p* < 0.05) among months of peak breeding season.

In the sperm during peak breeding season, mRNA expression levels of *Hdh-TEKT4* were found to be significantly different between two peak breeding seasons. The sperm during the first peak breeding season (May and June) showed a significantly higher expression of *Hdh-TEKT4* than that during the second peak breeding season (September and October) (Figure 10). However, no significant differences in mRNA expression of *Hdh-TEKT4* were observed between months in the first or the second peak breeding season. The percent sperm motility for sperm of these months also showed similar patterns (Figure 10).

### 3.11 Expression of *Hdh-TEKT4* mRNA and Percent Motility of Sperm of Pacific Abalone During Induced Spawning Activity Steps

**FIGURE 11:**
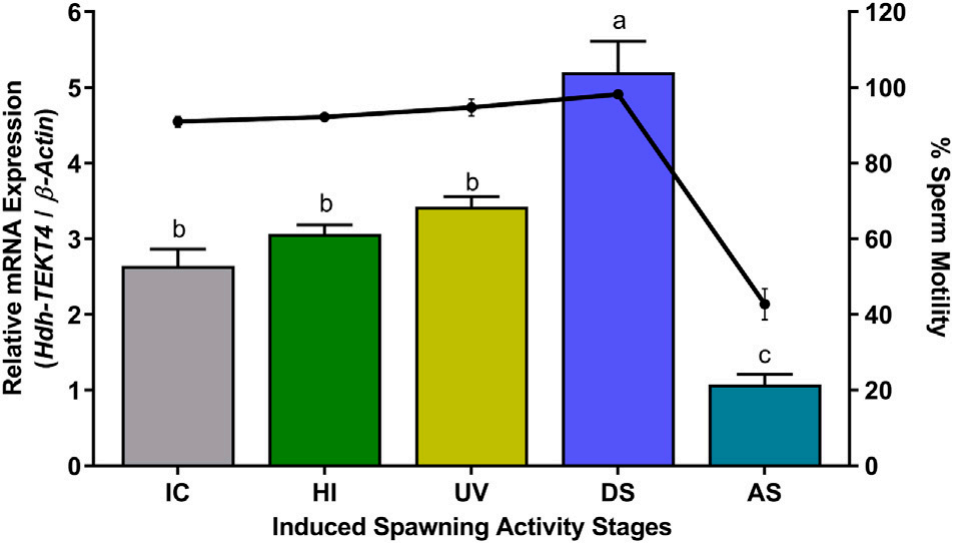
Expression levels of *Hdh-TEKT4* mRNA in sperm of Pacific abalone and percent sperm motility during different steps of induced spawning activities. Different letters above the bars indicate significant differences (*p* < 0.05) among steps of induced spawning. IC, initial control; HI, heat-induced; UV, UV-irradiated water-induced; DS, during spawning, AS, after spawning.

In different steps of induced spawning activity, a significantly higher mRNA expression of *Hdh-TEKT4* was observed in the DS stage than that in other stages (Figure 11). However, no significant changes in the mRNA expression of *Hdh-TEKT4* were observed among IC, HI, and UV steps. A marked decrease in the mRNA expression of *Hdh-TEKT4* was observed in the AS step. The percent sperm motility showed a similar pattern; significantly higher motility was observed in the DS stage compared to other steps. However, the sperm motility markedly decreased at the AS step from the DS step (Figure 11).

### 3.12 Expression of *Hdh-TEKT4* mRNA and Percent Sperm Motility in Different Cryopreserved Sperm of Pacific Abalone

**FIGURE 12:**
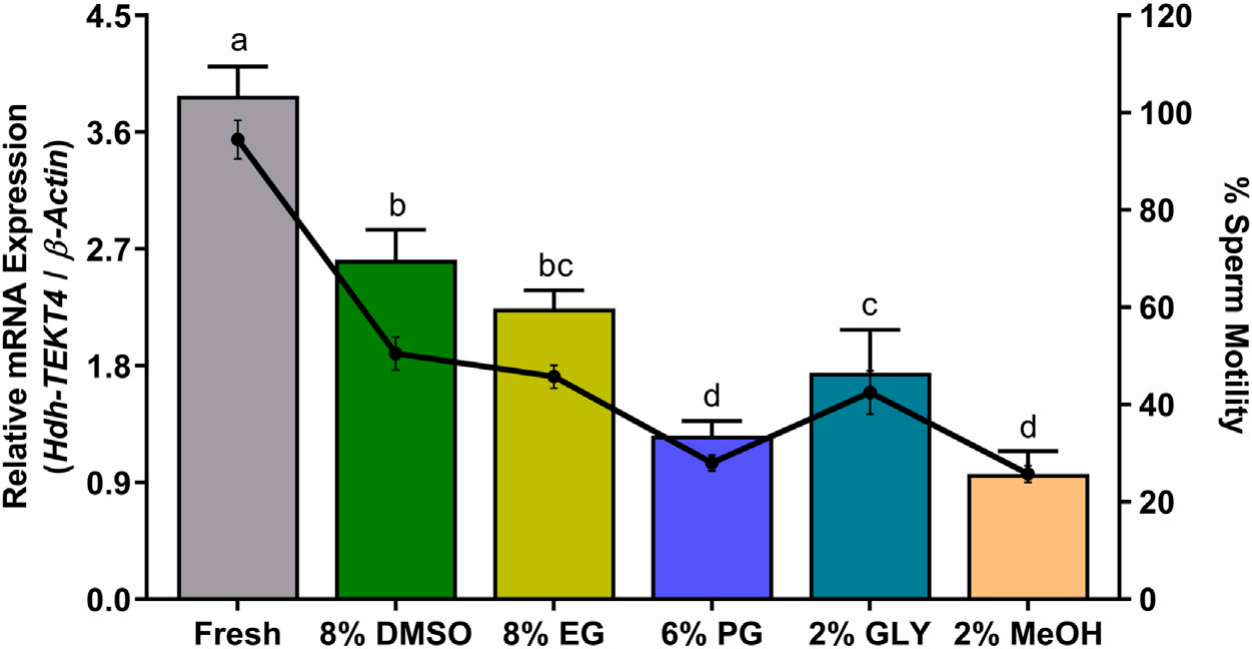
Expression levels of *Hdh-TEKT4* mRNA and percent sperm motility in fresh sperm of Pacific abalone and sperm cryopreserved in different cryoprotectants. Different letters above the bars indicate significant differences (*p* < 0.05) among different cryopreserved sperm. Fresh, fresh sperm; 8% DMSO, sperm cryopreserved in 8% DMSO; 8% EG, sperm cryopreserved in 8% ethylene glycol; 6% propanol glycol, sperm cryopreserved in 6% PG; 3% GLY, sperm cryopreserved in 3% glycerol; 2% MeOH, sperm cryopreserved in 2% methanol.

The levels of mRNA expression of *Hdh-TEKT4* and sperm motility of fresh (control) and cryopreserved sperm are shown in Figure 12. Fresh sperm showed significantly higher mRNA expression of *Hdh-TEKT4* than cryopreserved sperm. Sperm cryopreserved with 8% DMSO showed higher expression of *Hdh-TEKT4* than those cryopreserved with 2% MeOH. Sperm cryopreserved with 8% DMSO showed significantly higher mRNA expression of *Hdh-TEKT4* than those cryopreserved with 6% PG, 2% GLY, and 2% MeOH. However, no significant difference in the mRNA expression of *Hdh-TEKT4* was observed between 8% DMSO and 8% EG, between 8% EG and 2% GLY, or between 6% PG and 2% MeOH groups. A similar pattern was also observed for percent sperm motility. Fresh sperm showed significantly higher sperm motility than cryopreserved sperm. However, among cryopreserved sperm, 8% DMSO showed significantly higher motility than those sperm cryopreserved in other cryoprotectants.

### 3.13 ATP Levels and Motility of Sperm Under Different Experimental Conditions

The ATP levels of fresh sperm and sperm cryopreserved with different cryoprotectants during peak breeding season and percent sperm motility of same sperm are shown in Table 2. During two different peak breeding seasons, the ATP levels in fresh sperm (84.44 ± 2.9 and 72.10 ± 3.16 nM) showed significant variations. The ATP levels in cryopreserved sperm were significantly decreased than those in fresh sperm. The ATP levels also showed significant variations among sperm cryopreserved with different cryoprotectants. Sperm cryopreserved in 8% DMSO (50.20 ± 2.86 nM) and 6% PG (24.85 ± 1.35 nM) showed significantly higher and lower ATP levels, respectively, among sperm cryopreserved in different cryoprotectants. The sperm motility percent in the same sperm showed a similar pattern of decreasing trend as observed in the ATP content. Fresh sperm showed significantly higher motility than cryopreserved sperm. Among cryopreserved sperm, 8% DMSO showed significantly higher sperm motility than other cryopreserved sperms.

**TABLE 2:** ATP content and sperm motility (±SEM) in fresh and cryopreserved sperm of Pacific abalone.

	Fresh sperm (peak season)		Cryopreserved sperm				
	June	October	8% DMSO	8% EG	6% PG	2% GLY	2% MeOH
ATP (nM)	84.44 ± 2.9^a^	72.10 ± 3.16^b^	50.20 ± 2.86^c^	45.65 ± 3.43^c^	24.85 ± 1.35^e^	40.63 ± 3.13^cd^	29.92 ± 3.25^de^
Motility (%)	96.45 ± 0.36^a^	89.60 ± 0.85^b^	51.50 ± 1.70^c^	46.75 ± 1,18^cd^	28.00 ± 0.81^e^	43.50 ± 1.70^d^	26.50 ± 0.95^e^

Different superscript letters indicate significant differences (*p* < 0.05) among different values of ATP content and sperm motility.

## 4 Discussion

Tektins belong to a family of filament-forming proteins primarily expressed in the male germ-cell lineage. They are known to co-assemble with tubulins to form ciliary and flagellar microtubules. Tektins have been identified in various animals including humans (Xu et al., 2001; Wolkowicz et al., 2002; Matsuyama et al., 2005), mice (Iguchi et al., 1999; Cao et al., 2011; Takiguchi et al., 2011), silkworms (Ota et al., 2002), sea urchins (Norrander et al., 1992), and tropical abalones (Klinbunga et al., 2009). They are constitutively expressed proteins of microtubules in cilia, flagella, basal bodies, and centrioles (Norrander et al., 1998; Larsson et al., 2000). Tektins seem to be involved in the stability and structural complexity of axonemal microtubules. Several studies have reported the direct evidence for the importance of different Tektins in sperm. TEKT2-null male mice are infertile. TEKT2-null sperm displayed flagellar bending and reduced motility due to a disruption in the dynein inner arm (Tanaka et al., 2004). TEKT3-null sperm showed reduced motility, forward progression, and increased flagellar structural bending defects (Roy et el., 2009). TEKT4-null male mice are subfertile. TEKT4-null sperm have drastically reduced ATP levels and forward progressive velocity (Roy et al., 2007). Spermatozoa with Tektin-t deficient mice showed marked defects in mobility, frequent bending of the flagella, and structural disruption of the dynein inner arm (Tanaka et al., 2004). Tektin homolog in *Chlamydomonas* is diminished in mutant axonemes lacking inner arm dyneins (Yanagisawa and Kamiya, 2004). All these studies suggest that tektin plays a key role in sperm motility.

In this study, a full-length cDNA of *H. discus hannai* Tektin-4 (*Hdh-TEKT4*) was sequenced and characterized from the testis tissue of Pacific abalone. Hdh-TEKT4 protein contains a tektin domain (54-440 aa) with a nonapeptide signature sequence (RPNVELCRD), which is conserved in all members of the tektin family. The nonapeptide signature sequence, RPNVELCRD motif, was found in the Hdh-TEKT4 protein as RPGVDLCRD, with a replacement of asparagine and glutamic acid by glycine (position 367) and aspartic acid (position 369), respectively, in the specific nonapeptide sequence. Variants of this conserved nonapeptide motif have also been reported in *H. asinina* Ha-Tek-A1 (RPGVDLCRD), *Drosophila melanogaster* Tektin-A (RPNVENCRD), *Mus musculus* tektin-t (RSNVELCRD), *C. elegans* tektin (RPGIELCND), human tektin B1-like protein (RPNVEFCRD), and *Bombyx mori* testis specific tektin, BmTST (RPNVENCRD) (Ota et al., 2002; Amos, 2008).

*In situ* hybridization localization revealed that *Hdh-TEKT4* mRNA antisense probe expressed positive signals in the spermatid of abalone mature testis. It has also been reported that *TEKT4* mRNA expresses strong signals in the round spermatid of adult rat testis (Matsuyama et al., 2005). The gene ontology prediction analysis of Hdh-TEKT4 predicted this protein as the movement of cell or subcellular component protein.

A series of qRT-PCR analyses were performed in this study to observe the mRNA expression of *Hdh-TEKT4* in different experimental conditioned testis and sperm samples of Pacific abalone. The tissue distribution analysis revealed that *Hdh-TEKT4* was predominantly expressed in the testis tissue without any expression in the ovarian tissue. In testicular developmental or maturation stages, *Hdh-TEKT4* mRNA was expressed in all maturation stages of the testis, showing a significantly higher expression in the ripening stage. It was chronologically upregulated during testicular development. Furthermore, the mRNA expression of *Hdh-TEKT4* in the testis of Pacific abalone during broodstock conditioning exposed to effective accumulative temperature (EAT) showed a similar expression pattern as observed in testicular developmental stages. In abalone hatchery, broodstock conditioning is performed by exposing abalones to EAT at 18°C (when normal water temperature exists at about 7°C) for about 4 months for early gonadal maturation to change the time of spawning (Sukhan et al., 2021b). With the progress of EAT, abalone gonads gradually develop. When EAT reaches 1,500°C, gonads of Pacific abalone reach a higher maturity. Sperm maturity is the development of spermatozoa from nonfunctional gametes to mature spermatozoa, which are fully capable of vigorous motility and fertilization (Morisawa and Morisawa, 1986; Miura et al., 1992). The expression of *Hdh-TEKT4* in the testis of EAT-exposed Pacific abalone was also gradually upregulated with the increase in EAT, that is, with the progress of testicular maturation. Most advanced mature testis at EAT 1,500°C showed a significantly higher mRNA expression of *Hdh-TEKT4*. These results suggest that the *Hdh-TEKT4* gene might be involved in advanced stages of spermatogenesis when sperms become fully mature with higher motility. The expression of *TEKT4* in human (Matsuyama et al., 2005) and *Ha-TekA1* (Klinbunga et al., 2009), identical to *TEKT4* in tropical abalone, showed similar patterns of mRNA expression during testicular developmental stages.

In the sperm of peak breeding season, the mRNA expression of *Hdh-TEKT4* showed significant variations among two peak breeding seasons. The first peak breeding season (May and June) showed a significantly higher expression than the second peak breeding season (September and October). In this case, sperm motility was also determined. It was observed that sperm motility also showed significant differences between the two peak breeding seasons. This result may suggest that *Hdh-TEKT4* has a direct relation to sperm motility. Furthermore, the mRNA expression of *Hdh-TEKT4* was analyzed in the sperm of Pacific abalone during different steps of induced spawning activity. Induced spawning of Pacific abalone can be performed using fully mature male abalone when sperms reach maximum maturity having the best motile sperm (Uki and Kikuchi, 1984; Sukhan et al., 2021b). It is known that the sperm motility of aquatic animals is activated at the maximum level during the spawning stage when the sperm is released in an aquatic environment (Nichols et al., 2021). Once sperm motility is activated in maximum, the sperm subsequently loses its motility within a limited time (Linhart et al., 2008; Alavi et al., 2019; Boulais et al., 2019). It was observed that *Hdh-TEKT4* showed a significantly higher mRNA expression during the spawning step when the sperm motility reached the maximum motility during spawning stages for Pacific abalone. It was then drastically downregulated after spawning. This study’s result also suggests that the *Hdh-TEKT4* gene is associated with sperm motility. A previous study has shown that the TEKT3 peptide is significantly more abundant in the spawning group of greenlip abalone, *H. laevigata*, than in the group of abalones with failed spawning (Mendoza-Porras et al., 2014).

During the sperm cryopreservation process, sperms are exposed to toxic cryoprotectants. Changes of extreme temperature can cause physical damages to the sperm, such as decreases of plasma membrane integrity, acrosomal integrity, mitochondrial membrane potential, and motility (Stanic et al., 2000; Soni et al., 2019; Hossen et al., 2021a; 2021b). The freeze–thaw process of cryopreserved sperm can damage the sperm and affect their fertilization capacity by damaging their cell membrane, acrosomes, and DNA, and by undermining sperm motility (Centola et al., 1992). It can also alter the mRNA stability and abundance levels of protein and mRNA transcripts (Hezavehei et al., 2018). This study showed that mRNA transcript levels of *Hdh-TEKT4* in different cryopreserved and fresh sperm were significantly decreased in cryopreserved sperm than in fresh sperm. However, among different cryopreserved sperm, those cryopreserved with 8% DMSO and 8% PG showed significantly higher mRNA expression of *Hdh-TEKT4* than those cryopreserved with other cryoprotectants. The transcript level of *Hdh-TEKT4* in different cryoprotectant groups showed the following order: 8% DMSO > 8% EG > 2% GLY > 6% PG > 2% MeOH. This study’s finding coincided with that of other studies reporting that sperm cryopreservation could alter the mRNA-protein interaction and make mRNA more susceptible to degradation (Flores et al., 2011). The mRNA expression of *TEKT2* in cryopreserved human sperm is significantly reduced than that in fresh sperm (Alshawa et al., 2019). In addition, it has been reported that TEKT3 and TEKT4 are essential for coordinated and progressive sperm motility in mice (Roy et al., 2007; Roy et al., 2009). TEKT-t deficit male mice show impaired sperm motility than wild-type mice (Tanaka et al., 2004). The expression of *TEKT2* is significantly lower in the sperm of Murrah buffalo with lower motility than in the sperm with higher motility (Xiong et al., 2018). A lower expression of *TEKT4* mRNA transcript has also been reported in the spermatozoa of cyprinid fish with low motility than in the spermatozoa with high motility (Hu et al., 2017). In this study, it was observed that *Hhd-TEKT4* mRNA showed a significantly lower expression in sperm with low motility than in sperm with high motility during the peak breeding season, during spawning activity steps, and in different cryopreserved sperm (Figures 10–12).

The ATP levels in fresh sperm showed significant variations between the two peak seasons. Among different cryopreserved sperm, the ATP levels also showed significant variations. The ATP levels in sperm during peak breeding seasons and in different cryopreserved sperm showed a trend of variation similar to that observed for sperm motility and mRNA expression of *Hdh-TEKT4* in the same sperm sample. It has been reported that the motility and ATP content of oyster sperm can be significantly affected by the freeze–thaw process of cryopreservation (Demoy-Schneider et al., 2018). A significant decrease in the ATP content in the cryopreserved sperm of Pacific oyster than in fresh sperm has been reported (Suquet et al., 2010). The energy required for motility is derived from ATP. Sperm motility is highly correlated with the sperm ATP level (Mita and Nakamura, 1998). The ATP levels were significantly reduced in TEKT4-null mice sperm than in wild-type mice sperm (Roy et al., 2007). In this study, it was also observed that sperm ATP levels were lower in sperm with low motility. In addition, lower mRNA expression levels of *Hdh-TEKT4* were observed in sperm having lower ATP levels and lower motility.

In summary, *Hdh-TEKT4* was cloned from a molluscan species, Pacific abalone, which is related to sperm motility. *Hdh-TEKT4* was predominately localized in the spermatid, suggesting that it might act as an axonemal component. The results of mRNA expression analysis of *Hdh-TEKT4* in different experimental fresh and cryopreserved sperm and sperm motility analysis suggest that it is expressed at a higher level when the sperm motility and ATP level is higher. It could be concluded that *Hdh-TEKT4* may regulate the motility of the Pacific abalone sperm.

## Data Availability

The datasets presented in this study can be found in online repositories. The names of the repository/repositories and accession number(s) can be found at: https://www.ncbi.nlm.nih.gov/, MZ265399.
