# Supplementary material for: Hdh-Tektin-4 Regulates Motility of Fresh and Cryopreserved Sperm in Pacific Abalone, Haliotis discus hannai
Source: Front Cell Dev Biol. 2022 Apr 25;10:870743. doi: 10.3389/fcell.2022.870743 (PMC9081794; doi:10.3389/fcell.2022.870743)
Supplement: Supplementary file 1 [file DataSheet1.docx]

***Hdh-Tektin-4* Regulates Motility of Fresh and Cryopreserved Sperm of Pacific Abalone, *Haliotis discus hannai***

**Supplementary Figures**


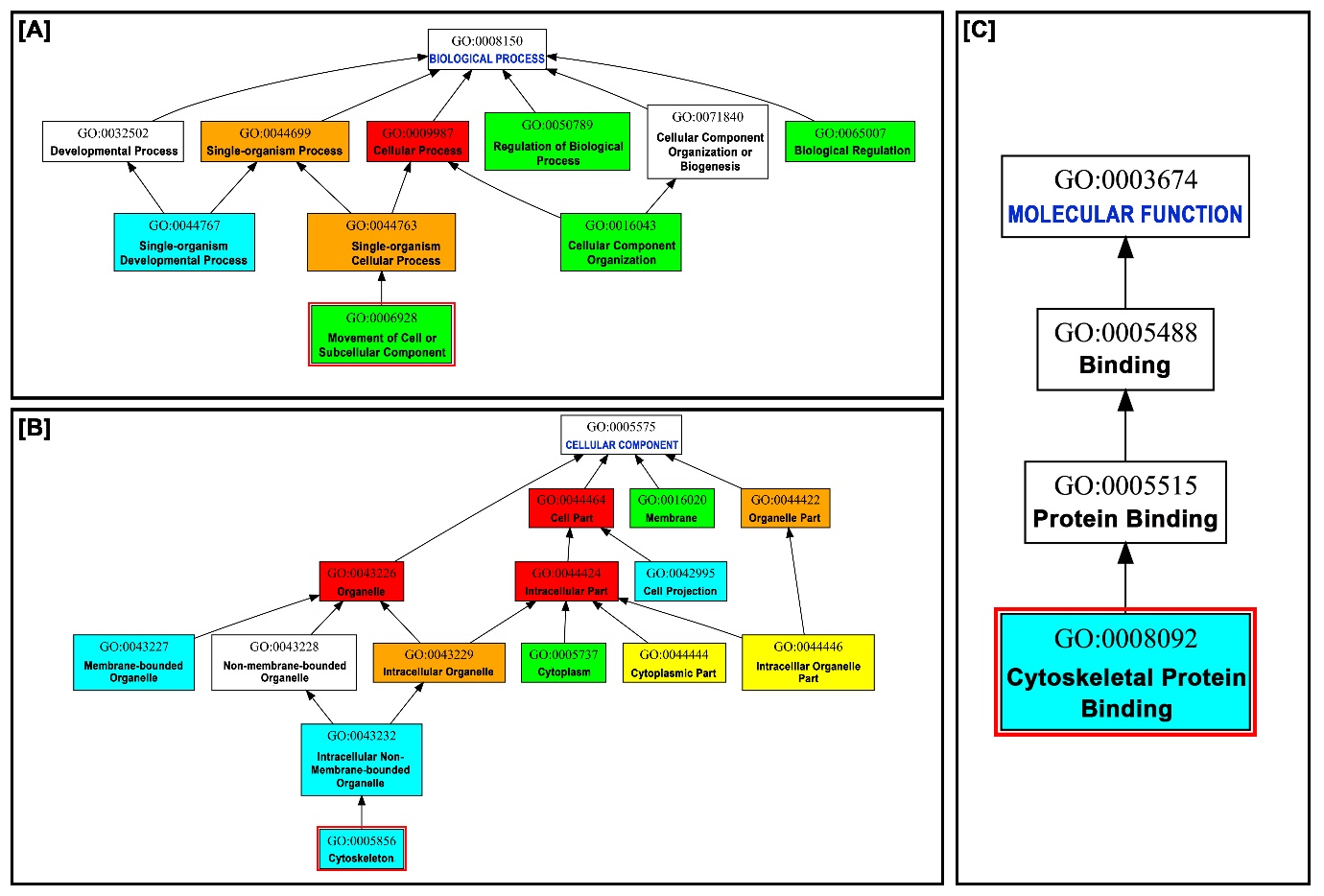


**S Figure 1.** Gene ontology (GO) of Hdh-TEKT4 gene of *H. discus hannai*. Figure illustrated three component of gene ontology, [A] biological process, [B] cellular component and [C] molecular function. Biological process revealed Hdh-TEKT4 as movement of cell or subcellular component protein.


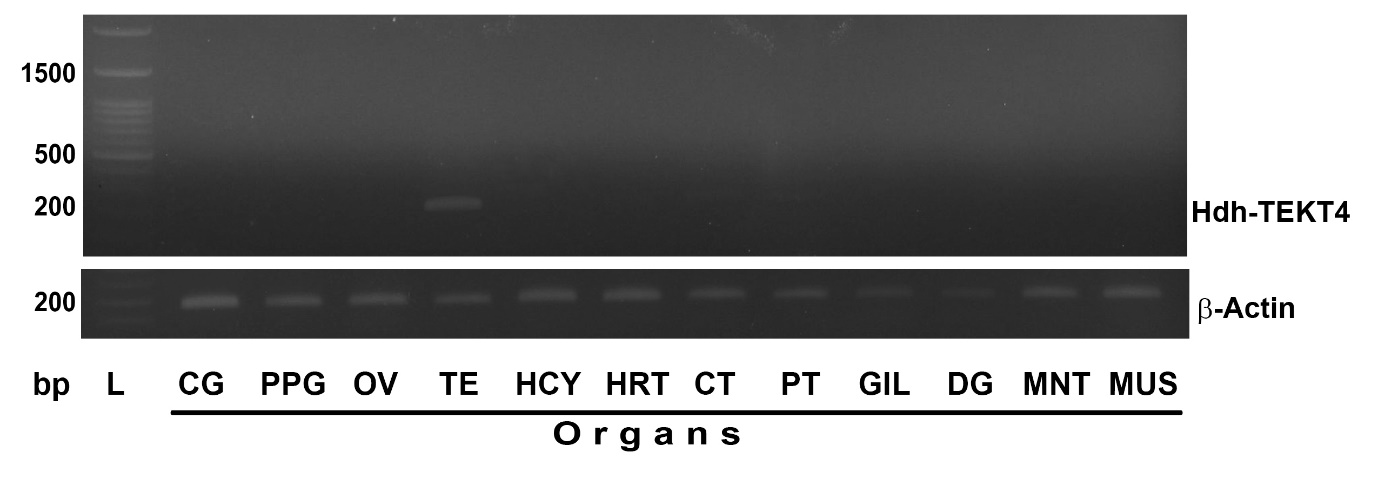


**S Figure 2.** Gene expression analyses of *Hdh-TEKT4* in various tissues of *Haliotis discus hannai* and *β-Actin* gene as reference gene. Lane L, 10000 bp DNA marker; CG, cerebral ganglion; PPG, pleuropedal ganglion; OV, ovary; TE, testis; HCY, hemocyte; HRT, heart; CT, cephalic tentacle; PT, pleuropedal tentacle; GIL, gill; DG, digestive gland; MNT, mantle; MUS, muscle


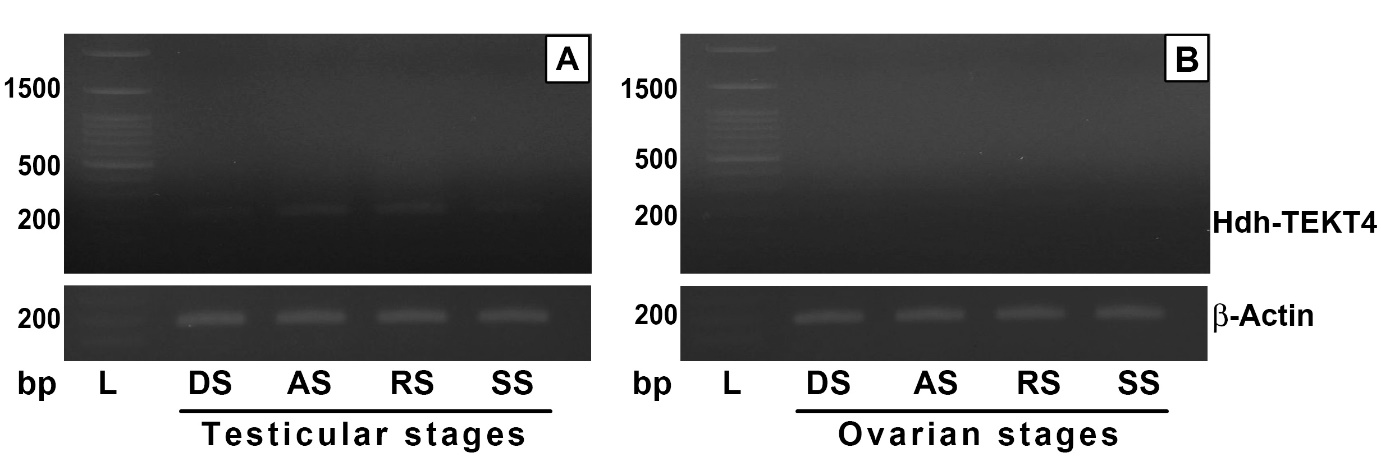


**S Figure 3.** Gene expression analyses of *Hdh-TEKT4* in different gonadal developmental stages of *Haliotis discus hannai* and *β-Actin* gene as reference gene: [A] Testicular developmental and [B] Ovarian developmental stages. Lane L, 10000 bp DNA marker; DS, degenerative stage; AS, active stage; RS, ripening stage; SS, spent stage.
